# EPs^®^ 7630 Stimulates Tissue Repair Mechanisms and Modifies Tight Junction Protein Expression in Human Airway Epithelial Cells

**DOI:** 10.3390/ijms241311230

**Published:** 2023-07-07

**Authors:** Lei Fang, Liang Zhou, Žarko Kulić, Martin D. Lehner, Michael Tamm, Michael Roth

**Affiliations:** 1 Pulmonary Cell Research, Department of Biomedicine & Clinic of Pneumology, University and University Hospital Basel, CH-4031 Basel, Switzerland; 2 Preclinical Research and Development, Dr. Willmar Schwabe GmbH & Co. KG, D-76227 Karlsruhe, Germany

**Keywords:** acute respiratory tract infection, EPs^®^ 7630, airway epithelium repair, epithelial tight junction proteins, intracellular signaling

## Abstract

Airway epithelium repair after infection consists of wound repair, re-synthesis of the extracellular matrix (ECM), and tight junction proteins. In humans, EPs^®^ 7630 obtained from *Pelargonium sidoides* roots reduces the severity and duration of acute respiratory tract infections. The effect of EPs^®^ 7630 on tissue repair of rhinovirus-16 (RV-16) infected and control human airway epithelial cells was assessed for: (i) epithelial cell proliferation by manual cell counts, (ii) epithelial wound repair by “scratch assay”, (iii) ECM composition by Western-blotting and cell-based ELISA, and (iv) epithelial tight junction proteins by Western-blotting. EPs^®^ 7630 stimulated cell proliferation through cAMP, CREB, and p38 MAPK. EPs^®^ 7630 significantly improved wound repair. Pro-inflammatory collagen type-I expression was reduced by EPs^®^ 7630, while fibronectin was increased. Virus-binding tight junction proteins desmoglein2, desmocollin2, ZO-1, claudin1, and claudin4 were downregulated by EPs^®^ 7630. The RV16-induced shift of the ECM towards the pro-inflammatory type was prevented by EPs^®^ 7630. Most of the effects of EPs^®^ 7630 on tissue repair and regeneration were sensitive to inhibition of cAMP-induced signaling. The data suggest that EPs^®^ 7630-dependent modification of epithelial cell metabolism and function might underlie the faster recovery time from viral infections, as reported by others in clinical studies.

## 1. Introduction

The maintenance of airway epithelium integrity and its fast repair are key factors for defense against viral and bacterial infections of the respiratory tract. Inhaled particles or microorganisms may disrupt the structure of the epithelium, and incomplete repair resulted in lasting loss of function [1]. These lasting changes in the airway wall’s structure and function might impair the ability of the airways to protect against further infection or damage [1]. Loss of the epithelium structure increases the risk of viral infection for epithelial progenitor cells and sub-epithelial mesenchymal cells [2,3].

The structure and function of the airway epithelium as a barrier between tissues and inhaled air is controlled by the composition of the local extracellular matrix (ECM) and by epithelial cell tight junction proteins [4]. The local composition of the ECM is subject to constant degradation and de novo synthesis, which is largely regulated through various cytokines and growth factors modulated by rhinovirus (RV), such as the transforming growth factor (TGF)-β1 [5]. Importantly, the ECM contains latent TGF-β, which is activated upon tissue degradation and thereby controls the differentiation stage of the embedded cells without the need for *de novo* synthesis [6].

Reduced epithelial cell tight junctions are a general pathology characterizing mucosal inflammation [6]. Upon RV infection, epithelial cells secreted TGF-β1, IL-1β, TSLP, and basic fibroblast growth factor, which degraded the tight junction complexes between epithelial cells and thereby enabled the infection of sub-epithelial cell layers [7,8,9]. The expression of tight junction proteins, such as E-cadherin, was regulated by cyclic AMP (cAMP) in epithelial cells [10].

RV infection is the predominant cause of cough and exacerbation in children, the elderly, and patients with chronic inflammatory lung diseases [10,11]. RV infection triggered the transition of airway epithelial cells into mesenchymal cells by inducing TGF-β1 and loss of epithelium integrity [9,12]. Thus, RV-induced changes in ECM composition might lead to airway wall remodeling and inflammation. In asthma patients, RV infection upregulated TGF-β1 secretion, which supported viral replication [3]. Therefore, fast and complete repair of the airway epithelium after infection is an essential aspect of the frontline host defense. The effect of RV infection on the ECM and epithelial cell tight junction proteins is not well understood.

In clinical studies, EPs^®^ 7630 reduced the duration and severity of acute respiratory tract infections in children [13,14] as well as in adults [15,16,17,18]. Whereas most clinical studies were performed in acute bronchitis, EPs^®^ 7630 was also reported to be effective in patients with common colds, where it reduced symptoms and led to faster recovery compared to placebo [16,19]. Furthermore, EPs^®^ 7630 revealed a significantly higher response and full remission rate compared to the placebo in treatment of acute rhino sinusitis of presumably bacterial origin [20] and demonstrated better clinical and anti-microbial effects than amoxicillin in patients with acute bacterial rhino sinusitis [21]. The safety and tolerability of EPs 7630 from clinical trials were also shown to be favorable in both adults and children [22].

Anti-viral activities of EPs^®^ 7630 have been reported in vitro against a number of viruses causing respiratory tract infections such as RV16 [23,24], Influenza A virus [25,26], RSV [25], and various coronaviruses, including a potent activity against SARS-CoV-2 [27]. In addition, EPs^®^ 7630 exhibits immunomodulatory effects including the modulation of cytokine release in vitro [28,29], reduction of tissue inflammation in a rat in vivo model [30], and reduction in nasal chemokine levels in a clinical bacterial rhino sinusitis study [21].

Given the perceived high relevance of airway epithelial cell homeostasis in recovery from respiratory tract infections, the effect of EPs^®^ 7630 on the expression of epithelial cell tight junction proteins, cell differentiation, and wound repair in human bronchial epithelial cells was assessed.

## 2. Results

### 2.1. EPs^®^ 7630 Regulates Epithelial Cell Proliferation through cAMP and p38 MAPK

**Figure 1:**
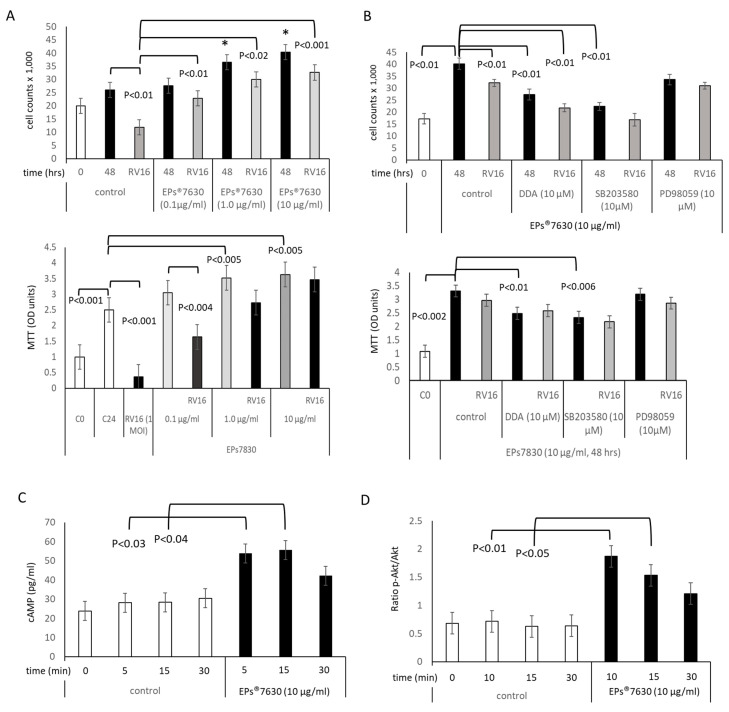

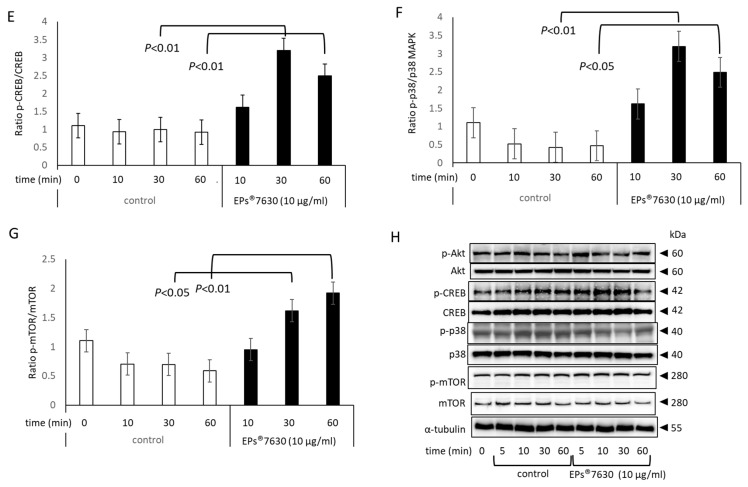
EPs^®^ 7630 stimulates epithelial cell proliferation and intracellular signaling. (**A**) EPs^®^ 7630 increased epithelial cell counts (48 h) and compensated cell loss of RV16-infected cells. * indicates significant difference compared to control 48 h. The effect of the different treatments was determined by direct cell count (upper panel) and by MTT assay (lower panel). (**B**) Role of signaling mediators on EPs^®^ 7630 induced cell proliferation. The effect of the different treatments was determined by direct cell count (upper panel) and by MTT assay (lower panel). (**C**) EPs^®^ 7630 stimulated the formation of intracellular cAMP. (**D**) Kinetic of EPs^®^ 7630 dependent shift of Akt to phosphorylated (p-)Akt. (**E**) Kinetic of EPs^®^ 7630 dependent shift of cyclic AMP response element binding protein (CREB) to p-CREB. (**F**) Kinetic of EPs^®^ 7630 dependent shift of protein-38 (p38) to p-p38 MAPK. (**G**) Kinetic of EPs^®^ 7630 dependent shift of mammalian target of rapamycin (mTOR) to p-mTOR. (**H**) Representative Western-blots used to generate the above shown bar charts of signaling proteins. Bars show mean ± S.E.M. of experiments performed in four different epithelial cell strains.

The effect of EPs^®^ 7630 on epithelial cells was tested in primary cells by direct cell count (Figure 1A upper panel) and by MTT assay in NuLi cells (Figure 1A lower panel). Both methods provided similar results, showing that in epithelial cells, EPs^®^ 7630 stimulated cell proliferation in a concentration-dependent manner (0.1–10 µg/mL) over 48 h (Figure 1A). RV16 infection (1 MOI: multiplicity of infection) over 48 h reduced cell numbers significantly, and this effect was counteracted by pre-incubation with EPs^®^ 7630 in a concentration-dependent manner (Figure 1A).

The signaling pathways regulating the mitogenic effect of EPs^®^ 7630 on epithelial cells were assessed by chemical inhibition of cAMP, Erk1/2, and p38, using direct cell counts in primary cells (Figure 1B upper panel) and in NuLi cells by MTT assay (Figure 1B lower panel). The EPs^®^ 7630 (10 μg/mL) -induced epithelial cell proliferation was significantly reduced in cells pre-incubated with either DDA or SB203580, but not by PD98059 (Figure 1B). In addition, the inhibition of either cAMP or p38 MAPK signaling furthered the negative effect of RV16 infection on cell counts (Figure 1B).

EPs^®^ 7630 (10 μg/mL) significantly increased the intracellular level of cAMP within 5 min, which declined thereafter (Figure 1C). Due to the short reaction time of cAMP, it was not possible to assess if RV16 infection modified the formation of cAMP. Subsequently, EPs^®^ 7630 (10 μg/mL) induced the phosphorylation of the cAMP substrates Akt, which peaked at 15 min (Figure 1D). Furthermore, the cAMP response element-binding protein (CREB) was phosphorylated, reaching a maximum within 30 min (Figure 1E). EPs^®^ 7630 also activated the phosphorylation of p38 MAPK over 60 min (Figure 1F). In the same samples, mTOR was phosphorylated after 30 min (Figure 1G). Representative Western-blots of phosphorylation patterns of the signaling proteins are shown in Figure 1H. In contrast, neither JNK-MAPK nor STAT3 (TGF-β signaling) expressions were affected by any condition. α-tubulin was used as and loading control indicators, respectively.

### 2.2. EPs^®^ 7630 Modifies the ECM Composition of Epithelial Cells

**Figure 2:**
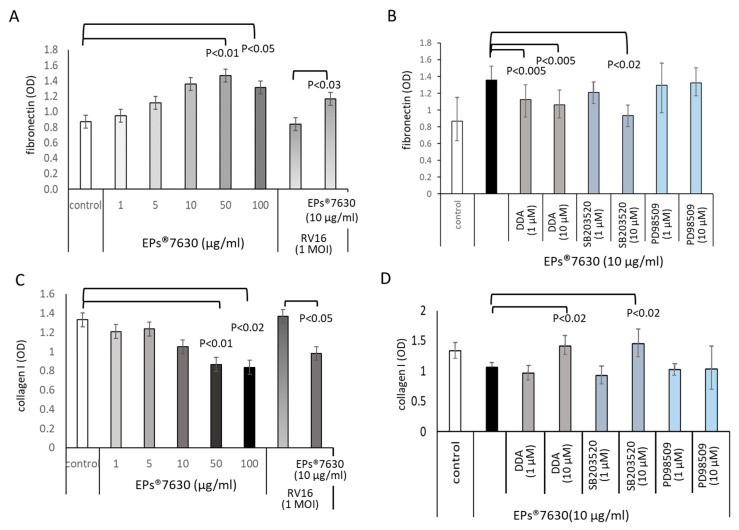
Effect of EPs^®^ 7630 on the expression of fibronectin and collagen type-I. (**A**) Cell-based ELISA analysis of fibronectin deposition induced by EPs^®^ 7630. (**B**) Role of cell signaling on EPs^®^ 7630-induced fibronectin deposition. (**C**) Inhibitory effect of EPs^®^ 7630 on collagen type-I deposition. (**D**) Collagen type-I expression influenced by different cell signaling inhibitors. Bars show mean ± S.E.M. of n = 4 cell lines.

Increased cell numbers in EPs^®^ 7630 treated cells were paralleled by increasing deposition of fibronectin, as shown by cell-based ELISA for fibronectin (Figure 2A). The stimulating effect of EPs^®^ 7630 on fibronectin deposition was concentration dependent and also occurred in RV16-infected cells (Figure 2A). RV infection did not affect the synthesis of fibronectin (Figure 2A). Inhibiting cAMP by DDA pre-incubation or by the p38 MAPK inhibitor SB203520 reduced the EPs^®^ 7630-stimulated deposition of fibronectin (Figure 2B). In contrast, the inhibition of Erk1/2 MAPK had no effect (Figure 2B).

Collagen type-I expression was detected in untreated cells, which was down-regulated by EPs^®^ 7630 (Figure 2C). RV16 infection upregulated the deposition of collagen type-I and was reduced when cells were pre-treated with EPs^®^ 7630 (Figure 2C). An inhibitory effect of EPs^®^ 7630 was observed for collagen type-I deposition in a concentration-dependent manner, as shown by cell-based ELISA Figure 2D. Blocking either cAMP or Erk1/2 MAPK also counteracted the inhibitory effect of EPs^®^ 7630 on collagen type-I deposition (Figure 2D).

### 2.3. EPs^®^ 7630 Modifies Epithelial Cell Tight Junction Protein Expression

**Figure 3:**
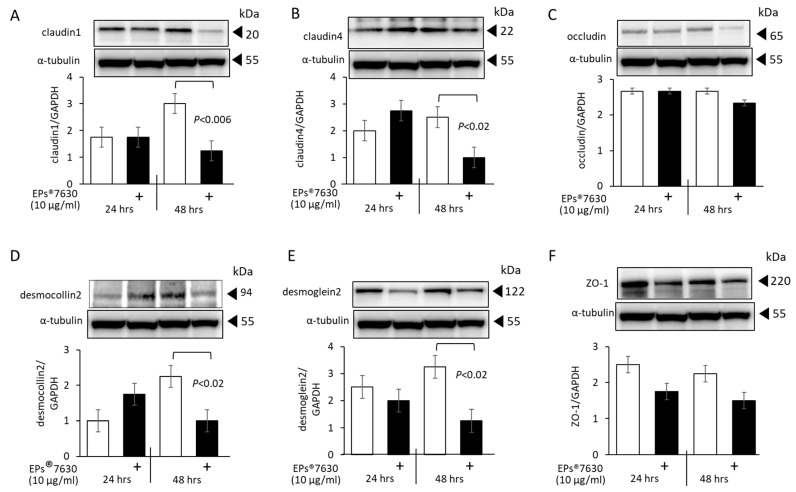
EPs^®^ 7630 modifies the expression of tight junction proteins and cell differentiation. Representative Western-blots and the image analysis of four Western-blots are presented as bar charts of the mean ± S.E.M. for: (**A**) claudin1, (**B**) claudin4, (**C**) occludin, (**D**) desmocolin2, (**E**) desmoglein2, and (**F**) ZO-1. The loading control, α-tubulin, was the same for all six tight junction proteins. Statistics were calculated using Student’s *t*-test.

Primary epithelial cells showed distinct cell line specific expression pattern of different tight junction proteins, while NuLi cells expressed all of them. Thus, NuLi cells were used to assess the effect of EPs^®^ 7630 on the expression of tight junction proteins (Figure 3).

As shown in Figure 3A, claudin1 was upregulated in native cells over 48 h, and this was reduced in cells treated with EPs^®^ 7630. The level of claudin4 increased initially (24 h) but was reduced after 48 h in cells treated with EPs^®^ 7630 (Figure 3B). The expression level of occludin was not significantly affected by EPs^®^ 7630 (Figure 3C). Desmocollin2 was upregulated over 48 h in untreated cells and was significantly reduced by EPs^®^ 7630 (Figure 3D). Desmogelin2 was also downregulated in EPs^®^ 7630 treated cells at both time points and became significant at 48 h (Figure 3E). The expression of ZO-1 was reduced by EPs^®^ 7630 at both time points compared to untreated cells, but did not achieve significance (Figure 3F).

### 2.4. EPs^®^ 7630 Increases Epithelial Cell Differentiation and Host Defense

**Figure 4:**
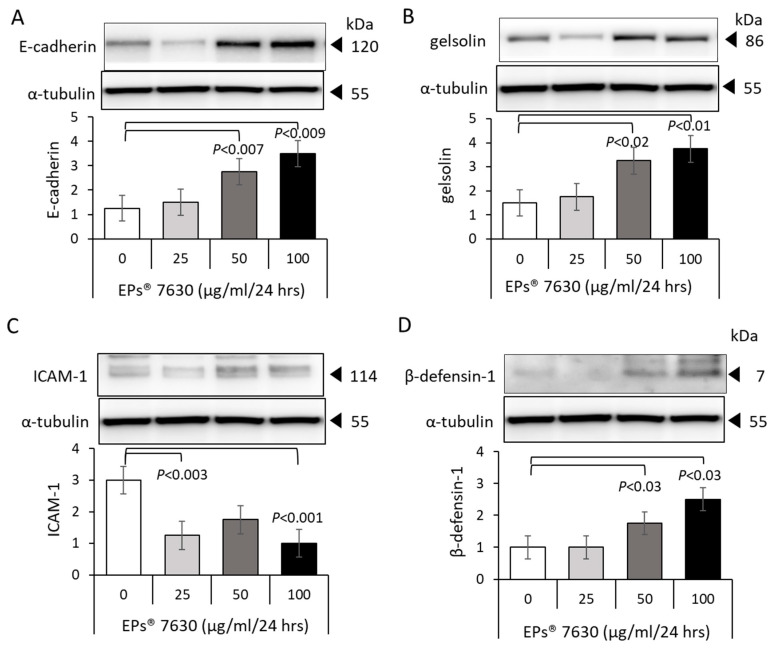
EPs^®^ 7630 modifies the expression of tight junction proteins and cell differentiation. Representative Western-blots and subsequent image analysis of four independent experiments are shown for (**A**) E-cadherin, (**B**) gelsolin, (**C**) ICAM-1, and (**D**) β-defensin-1. The loading control, α-tubulin, was the same for all five tight junction proteins. Bar charts represent mean ± S.E.M., and *p*-values were calculated using Student’s *t*-test.

Confirming our earlier data, EPs^®^ 7630 increased the expression of E-cadherin using epithelial cells in a concentration-dependent manner (Figure 4A). Similar to this, the expression of gelsolin was also stimulated by EPs^®^ 7630, as shown in Figure 4B. In regard to RV infection and host defense, EPs^®^ 7630 downregulated one of the major RV receptors, ICAM-1, in a concentration-dependent manner (Figure 4C). In contrast, the expression of β-defensin-1 was significantly upregulated over 24 h by EPs^®^ 7630 (Figure 4D).

### 2.5. EPs^®^ 7630 Improves Wound Repair

**Figure 5:**
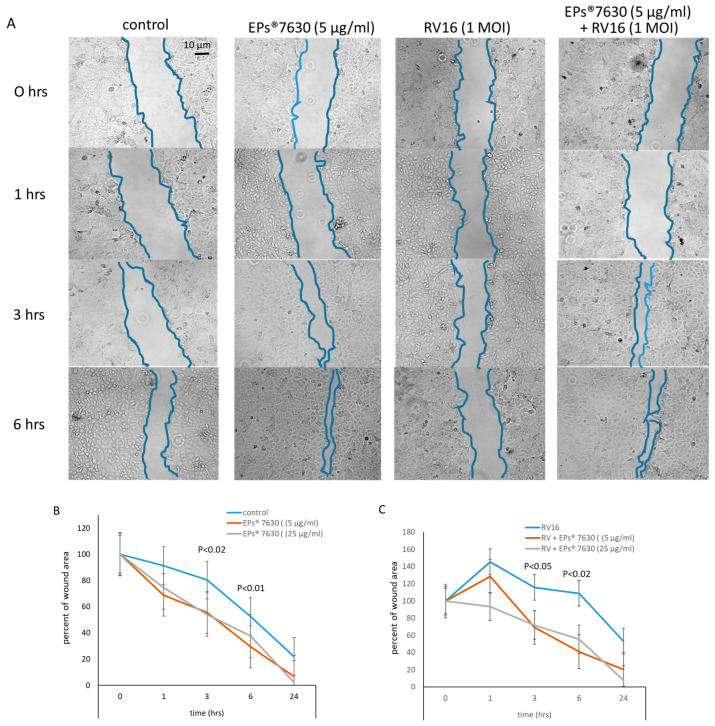
Effect of EPs^®^ 7630 on wound repair in human primary epithelial cells. (**A**) Representative phase-contrast photographs and measurement of wound area (area in between the two blue lines) over 6 h. Similar results were obtained in 2 additional cell lines. (**B**) Wound repair kinetic of naïve cells over 24 h. (**C**) Wound repair kinetic of RV16-infected cells over 24 h. Data points represent the mean ± S.E.M. of three cell lines; each cell line and time point were measured in duplicate experiments. *p*-values indicate significant differences comparing untreated to EPs^®^ 7630 pre-treated cells.

A scratched wound was generated in a confluent epithelial cell layer using a 10 µL pipette tip after the cells were pre-treated with EPs^®^ 7630 (in 0.35% ethanol-PBS, 5 µg/mL, and 25 µg/mL, 30 min), or vehicle-treated (0.35% ethanol in PBS), in the presence and absence of 1 MOI RV16. The size of the wound was monitored by phase-contrast microscopy over 24 h, and the area of the wound was measured (Figure 5A).

As shown in Figure 5B, naïve epithelial cells closed the wound within 8.5 h by 50%, while EPs^®^ 7630-treated cells showed a concentration-dependent significantly faster wound closure. When pre-treated with 5 µg/mL EPs^®^ 7630, 50% wound closure was achieved after 5.3 h, and when treated with 25 µg/mL EPs^®^ 7630, the wound was covered by 50% after 4.1 h. After 24 h, all wounds, except those of RV16-infected cells, were closed completely.

RV16-infected cells showed no wound closure over the first 6 h (Figure 5A); 50% wound closure was achieved after 24 h (Figure 5C). When pre-treated with EPs^®^ 7630 at 5 µg/mL, 50% wound closure was achieved after 5.2 h (Figure 5C). However, in cells pre-treated with 25 µg/mL EPs^®^ 7630, 50% wound closure was only achieved after 6.5 h (Figure 5C).

## 3. Discussion

**Figure 6:**
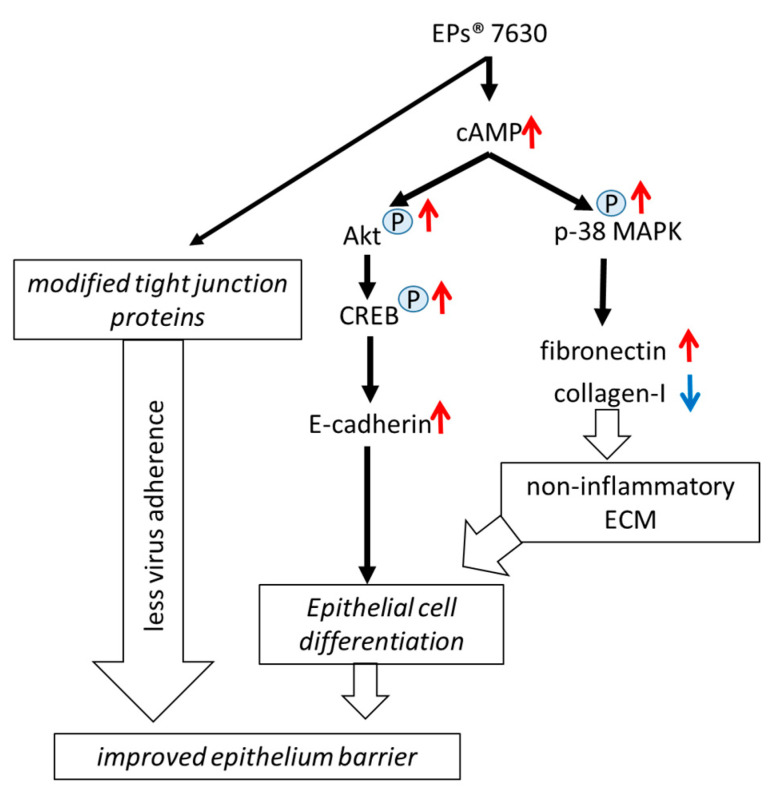
Summary of EPs^®^ 7630 induced cAMP-dependent regulation of epithelial barrier function and reduced expression of virus-adhering tight junction proteins. “P” in blue circle indicates phosphorylation of the corresponding signaling protein. Red arrow indicates upregulation and blue arrow indicates downregulation.

EPs^®^ 7630 has been documented to reduce the duration and severity of acute viral respiratory tract infections in patients with chronic inflammatory lung diseases. This beneficial effect of EPs^®^ 7630 was attributed to the prevention of virus attachment and replication in host cells [23,25,27]. The data presented in this study demonstrated that EPs^®^ 7630 might have additional properties on the function of host epithelial cells, which improve their differentiation and regeneration; a summary of the results is presented in Figure 6. These aspects are relevant to the post-viral recovery of the airway tissue [31].

The airway epithelium is the first line of defense of the airways and constitutes the entry point for viral pathogens. Viral infection reduces the integrity of the epithelial cell layer and it changes the composition of the local ECM embedding the cells. These effects can be mediated either directly by the virus or indirectly by inducing an inflammatory response in the host. RV infection has been shown to stimulate tissue remodeling and lead to the loss of epithelial integrity, at least in asthma [32,33]. The destruction of the epithelium by RV enhances the penetration of allergens leading to sub-epithelial inflammation [34]. Furthermore, in a co-culture model, RV infection of airway smooth muscle cells increased the replication of the virus in the epithelial cells of asthma patients [35]. Interestingly, RV infection was reported to stimulate airway wall remodeling through the induction of TGF-β1 [5] or by a member of the tumor necrosis factor family (LIGHT) and IL-1β [9]. RV-induced epithelial to mesenchymal transition (EMT) was dependent on changes in the composition of the ECM [12]. However, in this study, EPs^®^ 7630 did not modify the activity of the TGF-β-signal transducer STAT3, but activated Erk1/2 and cAMP, which is in line with earlier reports.

In regard to the intracellular signaling in EPs^®^ 7630 treated epithelial and immune cells, Erk1/2 and p38 MAPK were indicated as the major and early signaling proteins [24,36]. In the present study, the proliferation-stimulating effect of EPs^®^ 7630 in epithelial cells was dependent on the activation of cAMP in epithelial cells. In line with this finding, cAMP activation by forskolin inhibited TGF-β signaling and enhanced epithelial cell differentiation and proliferation [37]. In this study, EPs^®^ 7630 increased cAMP within minutes, and the inhibition of this mechanism by DDA disrupted all the above-described improvements in epithelial cell function.

RV infection caused the loss of cell-cell adhesion in the epithelium [38,39], and the loss of epithelial cell tight junctions as a consequence of infection had also been reported in children with asthma [40]. However, the nature of this loss of epithelial cell adhesion was not determined in detail. The above-described experiments might indicate that EPs^®^ 7630 might help to re-establish specific epithelial cell–cell tight junctions, which contribute to anti-viral defense and explain the faster post-viral infection recovery. This study provides the first evidence that EPs^®^ 7630 improves the formation of tight junctions between epithelial cells by upregulating three tight junction proteins 1: claudin4, desmocollin2, and desmoglein2.

The loss of claudin4 by influenza virus infection was linked to the breakdown of alveolar epithelial barrier function in humans [41] and was also shown for epithelial cell HIV infection in vitro [42]. Desmocollin2 is a member of the desmosomal cadherin family and thus regulates tight junctions. *Coxsackievirus* infection degraded desmocollin2, which resulted in tissue degradation and inflammation in cardio-myocytes [43]. Desmocollin2 is linked to the cell-cell adhesion function of fibronectin and in this context prevented anoikis [44]. Fibronectin was essential to maintain the epithelial cell’s function as a barrier [45]. The exact role of desmocollin2 in the context of infection should be further investigated. Thus, its upregulation by EPs^®^ 7630 might help cells survive viral infection and regenerate faster. Desmoglein2 is expressed on the apical side of epithelial cells and maintains the cell’s polarity [46]. However, together with other family members, desmoglein2 has also been described as a docking protein for viruses that cause acute respiratory distress syndrome [47,48]. The net effect of desmoglein2 induction by EPs^®^ 7630 might be enhanced epithelial differentiation, but the role of this effect in regard to virus infection has to be further studied.

The structure and barrier function of the epithelium are controlled by the composition of the ECM, which undergoes a rapid turnover of up to 10% daily [49]. RV infection during early life modified the composition of the ECM through the inhibition of degrading enzymes [50]. This may explain the upregulating effect of RV infection on fibronectin, perlecan, and collagen type-IV [5]. In the context of viral infection, RV increased the remodeling of airway smooth muscle cells and fibroblasts [2]. In the context of tissue regeneration and repair, fibronectin supports epithelial cell proliferation and wound repair [51]. Therefore, the observed increase in fibronectin in cells treated with EPs^®^ 7630 may contribute to the faster recovery documented in clinical studies [13,52]. In addition, EPs^®^ 7630 induced fibronectin might help to reduce bacterial infection of epithelial cells [53].

In this study, EPs^®^ 7630 improved the recovery of epithelial cells after experimental wounding in both control epithelial and RV16-infected epithelial cells. These effects of EPs^®^ 7630 might explain the reduced duration of symptoms in patients with RV infection [13,14,16,19]. These findings further strengthen the hypothesis that the maintenance of epithelial cell tight junctions and its interaction with the local ECM is an important factor to protect from or reduce the spreading of viral infection [54].

**Figure 7:**
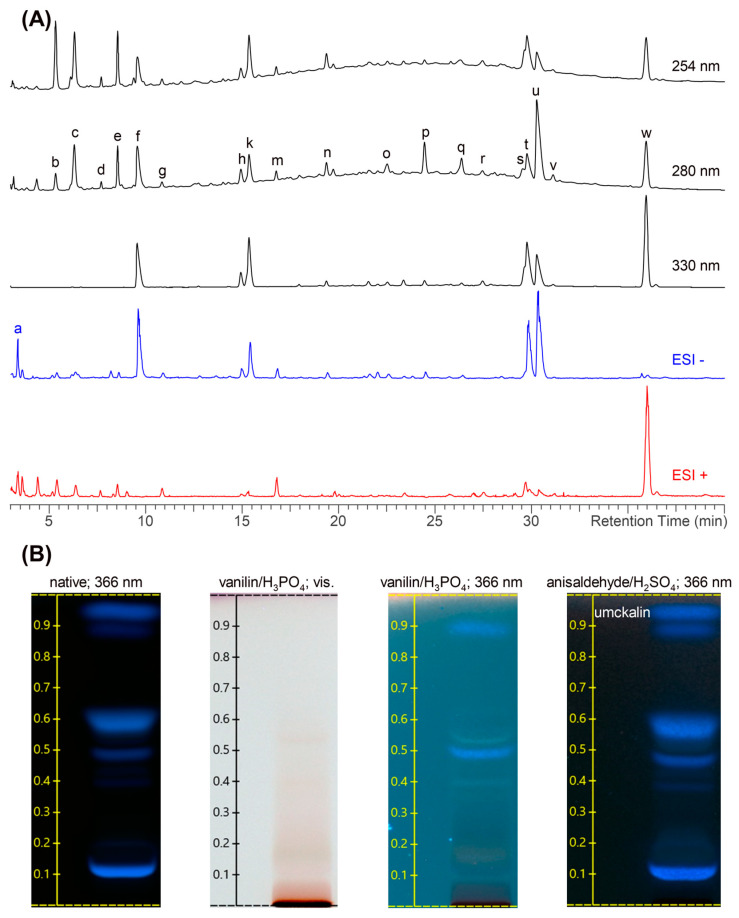
(**A**) LC-UV-HRMS fingerprint of EPs^®^ 7630 detected at different UV wavelengths and in negative and positive ion modes as annotated. The substance assignments a-w are given in Table 1. The prodelphinidins are visible at 254 nm and 280 nm as a bulge between ca. 12 and 38 min. (**B**) HPTLC fingerprint of EPs^®^ 7630 native and with different staining reagents as annotated, detected at visible light and 366 nm, respectively. Umckalin is the uppermost spot at an R_f_ of ca. 0.95. The dark spot at the start (R_f_ = 0) represents the prodelphinidins.

**Figure 8:**
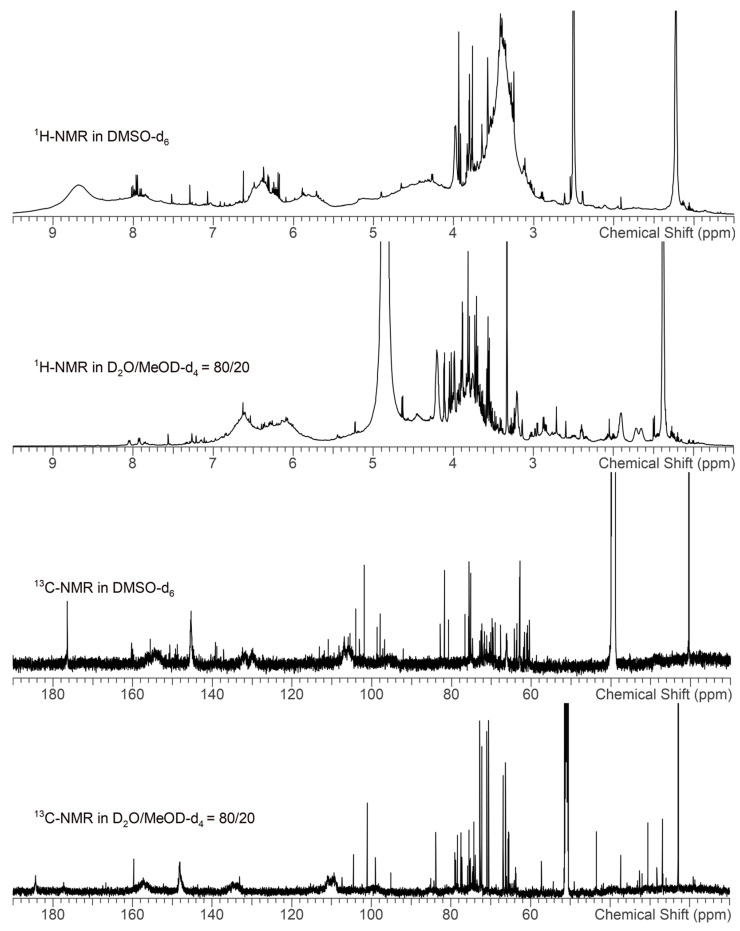
^1^H- and ^13^C-NMR fingerprints of EPs^®^ 7630 in two different solvents as annotated. The prodelphinidins have broad signals due to the oligomeric nature and the manifold structures of the substance class. In contrast, the benzopyranons and a carbohydrate portion have sharp signals, as characteristic for small molecules. In DMSO-d_6_, the OH groups of the prodelphinidins are visible as broad signals in the ^1^H spectrum between 7 and 9 ppm. These signals are absent in the ^1^H spectrum measured in D_2_O/MeOD-d_4_ because the prodelphinidin OH protons exchange with the deuterons of the solvent.

The identification of the defined active constituents in EPs^®^ 7630 was beyond the scope of this study. As an herbal extract, EPs^®^ 7630 has a complex composition with carbohydrates, minerals, peptides, purine derivatives, highly substituted benzopyranones, and oligo- and polymeric pro-delphinidins, as described earlier [24,55,56] and shown in the phytochemical characterization presented in Figure 7 and Figure 8. Pro-delphinidins belong to the compound class of pro-anthocyanidins, which have been shown to affect the tight junctions of the epithelium in various organs [57,58]. Pro-anthocyanidins were also identified as active constituents contributing to the anti-bacterial and anti-viral effects of EPs^®^ 7630 [53,59]. In addition, anthocyanins from another source prevented EMT of alveolar epithelial cells and upregulated the expression of E-cadherin in epithelial cells, thereby improving their differentiation and the formation of tight junctions [60]. Although literature data suggest relevant contributions of the polyphenol constituents in EPs^®^ 7630, the overall effect of the extract, as assessed in our models, is likely a combination of a multitude of different constituents with individual activities.

The limitation of this study includes the lack of information regarding the effect of EPs^®^ 7630 on the interaction between epithelial and mesenchymal cells before and after RV infection. Furthermore, it is difficult to prove that a specific component of the ECM, such as fibronectin, lowers the susceptibility of the host cells to viral or bacterial infection. The above-described effects of EPs^®^ 7630 have to be confirmed in patients with RV infection. It should be noted that such investigations cannot be performed in animal models due to species-specific tissue repair mechanisms [61]. Importantly, the composition of EPs^®^ 7630 makes it difficult to link the beneficial effect on tissue repair to one specific compound. Instead, the beneficial effects of EPs^®^ 7630 on tissue repair and recovery are likely to be the result of the specific combination of natural active substances contained within EPs^®^ 7630. Since the medicinal product contains the whole extract of EPs^®^ 7630 as the active pharmaceutical ingredient, the investigation of single components was not considered in this study.

In conclusion, the presented data showed that EPs^®^ 7630 modified intracellular signaling, ECM composition, and wound repair in human bronchial epithelial cells. The data obtained from cell lines and primary bronchial epithelial cells suggest that EPs^®^ 7630 improves tissue recovery after RV infection, which potentially contributes to the clinically documented recovery of patients from respiratory tract infections.

## 4. Materials and Methods

### 4.1. Human Bronchial Epithelial Cells

Six primary human bronchial epithelial cell lines were obtained from non-diseased tissue obtained from the resected lungs of cancer patients (3 males, 3 females, age range: 45–70 years), who were free of chronic inflammatory lung diseases (Pneumology Clinic, University Hospital Basel). All patients gave written informed consent for the use of tissue for translational scientific studies. The approval was obtained from the local ethical committee (Ethikkommission Nordwest- und Zentralschweiz, EKNZ) (PB_2019-00035).

Basic experiments on intracellular signaling pathways were performed in an immortalized human bronchial epithelial cell line: NuLi-1 (ATCC, Manassas, VA, USA).

All epithelial cells were grown in CnT-PR-A (CellnTech, Bern, Switzerland). Cells were used at confluence and treated with increasing concentrations of EPs^®^ 7630 (0.1–100 µg/mL) as described earlier [23,24].

### 4.2. EPs^®^ 7630

For all experiments, a sample from the production batch (EXCh. 878) of EPs^®^ 7630, a dried extract of *Pelargonium sidoides* DC. Roots (1:8-10), extraction solvent: ethanol 11% (*w*/*w*), was used. EPs^®^ 7630 was provided by Dr. Willmar Schwabe GmbH & Co. KG (Karlsruhe, Germany). A total of 80% of the roots used for the aforementioned production batch were collected from wild plant populations, while 20% were harvested from plantations in South Africa. The dried material was tested in an array of DNA-based and phytochemical methods to confirm the quality and identity of the herbal material. Pharmacognosy was performed by the quality control department of Dr. Willmar Schwabe GmbH and Co. KG. Voucher specimens of every lot were deposited in the Department of Pharmacognosy to be retained for ten years. The production batch used was characterized according to the Consensus statement on the Phytochemical Characterization of Medicinal Plant Extracts ConPhyMP [62]. The production batch was specified by the manufacturer to contain 28.6% total phenols and 1.93% total benzopyranons, with a sum of 0.75% for umckalin and umckalin sulfate. Chemical fingerprinting was carried out by three different methods, namely LC-UV-HRMS, HPTLC, and NMR, each with multiple detection parameters and with partial assignments of substances and substance classes according to literature data [55,62] and own interpretation of molecular masses (Figure 7 and Figure 8, Table 1).

The LC-UV-HRMS chromatograms were acquired on a Thermo Vanquish UHPLC coupled to a DAD and Thermo Orbitrap Fusion mass detector using a Waters Atlantis T3 (3 μM, 2 mm × 150 mm) column without a pre-column. Eluent A consisted of 2.5% (*v*/*v*) acetonitrile and 0.5% (*v*/*v*) formic acid in water. Eluent B consisted of 5% (*v*/*v*) water and 0.5% (*v*/*v*) formic acid in acetonitrile. At a flow rate of 0.2 mL/min, the gradient was as follows: from 0.0 to 10.0 min linear for 0% to 5% eluent B, from 10.0 to 65.0 min linear for 5% to 50% eluent B, from 65.0 to 66.0 min linear for 50% to 100% eluent B, from 66.0 to 71.0 min isocratic 100% eluent B column wash, from 71.0 to 72.0 min linear from 100% to 0% eluent B followed by 8 min equilibration period with 0% eluent B, resulting in a total run time of 80.00 min. UV detection wavelengths of 254, 280, and 330 nm and a column temperature of 40 °C were applied. The injection volume was 4 μL of a 5 mg/mL *P. sidoides* extract EPs^®^ 7630 dissolved in eluent A. HRMS chromatograms were acquired in positive and negative ion mode at a resolution of 30,000 and based peak assignment was performed using ACDLabs Spectrus Processor Software v2021.2.0.

HPTLC analysis was carried out using HPTLC 60 F254 (Merck, Darmstadt, Germany) plates. EPs^®^ 7630 samples were dissolved at a concentration of ca. 100 mg in 10 mL aqueous ethanol 10% (*v*/*v*) by sonication for ca. 10 min. An amount of 10 µL of the solution were spotted on the plates using a CAMAG Automatic TLC Sampler 4. Prior to analysis, a CAMAG Automatic Developing Chamber 2 was conditioned with the elution solvent for 10 min, and the humidity was subsequently equilibrated with a saturated MgCl_2_ ∙ 6 H_2_O solution for another 10 min. The HPTLC plates were conditioned in the chamber for 2 min prior to analysis. The elution solvent was composed as follows: ethyl acetate/methanol/water = 76/14/10 (*v*/*v*/*v*). The separation runs took approximately 30 min to reach 6 cm of separation length. After the run, the elution solvent was actively evaporated using an air stream for 5 min at room temperature, and the plates were sprayed with two different staining reagents, while one was left unstained. One staining reagent was prepared as follows: 20 mL deionized water, 35 mL 85% (m/m) phosphoric acid, 1 g vanillin (Sigma Aldrich, St. Louis, MO, USA), 2.5 mL 96% (*v*/*v*) ethanol were mixed until vanillin was completely dissolved, and subsequently the solution was diluted with deionized water ad 100 mL. The other staining reagent was prepared as follows: 0.5 mL p-Anisaldehyde (Sigma Aldrich), 85 mL methanol, 10 mL acetic acid, and 5 mL concentrated sulfuric acid. The staining was developed by placing the plates for ca. 2–3 min on a hot plate tempered to 125 °C. Visualization at visible light and under UV radiation at 366 nm wavelength was performed with a CAMAG TLC Visualizer 2 using CAMAG visionCATS v2.5 software.

NMR spectra were acquired using a Bruker Avance III HD System equipped with an inverse TCI Prodigy Cryoprobe. The Larmor frequencies for ^1^H and ^13^C were 600 MHz and 150 MHz, respectively. Samples were suspended at concentrations of 28 mg in 600 µL DMSO-d_6_ and 26 mg in 600 µL D_2_O/MeOD-d_4_ = 80/20 (*v*/*v*), respectively, and small amounts of solid residue were removed by centrifugation prior to measurement. For referencing, tetramethylsilane or trimethylsilylpropionic acid-d_4_ were used, respectively, and all spectra were acquired with a sample temperature of 25 °C. The 1D-^1^H spectra were acquired with 32 accumulated scans with a spectral width of 18 ppm, a transmitter frequency offset of 8 ppm, and a digital resolution of 64 k data points. The 1D-^13^C spectra were acquired with 16 k accumulated scans with a spectral width of 240 ppm, a transmitter frequency offset of 110 ppm, and a digital resolution of 64 k data points. The spectra were processed with an exponential window function with a line broadening of 0.3 Hz. The spectra were recorded and processed with Bruker Topspin software (v3.6pl7) and analyzed with ACD/Labs Spectrus Processor (v2021.2.0).

For all following cell assay experiments, EPs^®^ 7630 was dissolved in 10% ethanol (in phosphate buffered saline: PBS) and diluted to the desired concentrations with cell culture medium.

### 4.3. Cell Proliferation

Cell proliferation was determined by direct cell counting seeding cells at 10,000 cells/mL into 6-well plates. Cells were allowed to adhere overnight before being deprived of growth medium for 24 h. Following treatment with different conditions, cells were washed twice with phosphate buffer saline (PBS) and detached by trypsin/EDTA treatment. Trypsin was inhibited by addition of 2% serum, and cells were counted manually on a Neubaur chamber slide.

In addition, proliferation was determined by MTT assay in NuLi cells. Cells were seeded at 10,000/mL, and allowed to adhere overnight before being deprived of the growth medium for 24 h. After treatment, cells were incubated at 10 µL/well (96-well plate) for 4 h with 5 mg/mL MTT (3-[4,5-demethylthiazol-2-yl]-2,5 diphenyl tetrazolium bromide) for an additional 6 h under standard cell culture conditions. Next, the supernatant was removed, and cells were lysed overnight in 150 µL acidified isopropanol. The optical density (OD) was determined using a BioRad ELISA reader (iMark™ Microplate Absorbance Reader, Bio-Rad Laboratories AG, Cressier, Switzerland) at a wavelength of 540 nm.

### 4.4. Rhinovirus-16

RV-16 was purchased from ATCC (cat# VR-283, Manassas, USA). Sub-confluent epithelial cells (80%) were infected with 1 MOI RV-16 by 5 min centrifugation (200× *g*), as described earlier [24]. RV-16 infection was monitored by immunofluorescence microscopy using anti-RV-16 antibody (cat# 18758, QED-Bioscience Inc., San Diego, USA), followed by detection antibody labelled with FITC (Abcam, Cambridge, UK) [24].

### 4.5. Cyclic AMP

Cyclic AMP concentrations were detected by a commercial ELISA (cat# KGE002B, R&D Systems, Abingdon, UK) after lysing the cells at different time intervals (0, 5, 10, 30, and 60 min). The cell’s content of cAMP was determined following the instructions of the distributor.

### 4.6. Signaling Inhibitors

To assess the relevance of cAMP increase, adenylyl cyclase was inhibited by 30-min pre-incubation with 2’,3’-dideoxyadenosine (DDA, 10 μM, Thermo Fisher, Waltham, MA, USA), as described earlier [62].

Erk1/2 MAPK was inhibited by 30 min of pre-incubation with 10 μM PD98059, and p38 MAPK was inhibited by pre-incubation (30 min) with 10 μM SB203520 [24].

### 4.7. Western-Blotting

Cells were lysed in radio-immuno precipitation assay buffer (RIPA, Thermo Fisher Scientific, Waltham, USA), and the protein concentration was determined by BCA assay (Thermo Fisher). An equal amount of protein was denatured at 95 °C (5 min) before being applied to a gradient poly-acrylamide gel (4–12%, GenScript Biotech, Piscataway, NJ, USA), and size fractionated for 1 h (100 V). Proteins were transferred onto nitrocellulose membranes and incubated in blocking buffer (PBS-T = PBS + 0.05% Tween-20 + 5% bovine serum albumin). Membranes were incubated with the primary antibody overnight (4 °C) in blocking buffer, then washed 3 times with PBS-T, and incubated with a secondary HRP-labelled matching antibody for 1 h at room temperature. Protein bands were visualized by applying SuperSignal West Dura Substrate (Thermo Fisher) and documented using c300 Azure (Azure Biosystems, Dublin, CA, USA), as described earlier [63]. The antibodies and conditions are summarized in Table 2.

### 4.8. Wound Repair

Epithelial cell repair was modelled in isolated cells by generating a confluent cell layer, which was then scratched mechanically using the tip of a 10 µL Eppendorf pipette. The closing of the “wound” was monitored using a microscope over 24 h [64]. The area of the wound was measured by microscopy and computer-supported analysis of cell coverage (EVOS image station, Thermo Fisher). The wound area of untreated cells for each condition at time point zero was set to 100%, and the percentage of cell-free area was measured at subsequent time points (1, 3, 6, and 24 h after scratching), and the percentage of wound closure was calculated. Microscopic photographs were generated by EVOS M5000 (10 times using a phase-contrast filter.

### 4.9. Cell-Based ELISA for Collagens and Fibronectin

The deposition of collagens type-I/IV, fibronectin, and expression of E-cadherin was determined on the surface of confluent epithelial cell layers grown in 96-well plates (Sarstedt, Nümbrecht, Germany) by in vitro cell-based enzyme-linked immunosorbent assays (ELISA) as described earlier [65]. Primary antibodies are listed in Table 2 and were obtained from Bio-Techne Ltd., Abingdon, UK, Thermo Fisher, and BD Biosciences, Allschwil, Switzerland.

Cells were fixed by 2 × 5 min incubation with 2% paraformaldehyde in PBS, followed by 2 washes with PBS and an hour incubation with PBS containing 0.01% Tween-20 and 2% bovine serum albumin (blocking buffer). Plates were incubated with one of the primary antibodies overnight (4 °C, shaking) before being washed twice with blocking buffer and incubated with a species-specific horse radish oxidase labelled 2nd antibody (1 h, room temperature). Antibody binding was visualized after 3 washes with PBS by addition of 50 μL/well of substrate (TMBX) for 30 min. The color reaction was stopped by adding 50 μL/well 0.5 N HCl and adsorption was read at 450 nm by a BioRad ELISA reader [66]. Untreated cells were used as reference values.

### 4.10. Statistics

The null hypothesis was that EPs^®^ 7630 does not affect any of the above-named parameters. Data were analyzed by one-way ANOVA, or Student’s *t*-test. *p*-values were considered significant when <0.05. Data are presented as the mean ± S.E.M.

## Figures and Tables

**Table 1:** Substance assignments of the LC-UV-HRMS fingerprint in Figure 7.

Assignment	Substance				
a	Citric acid				
b	Adenosine 3′,5′-cyclic monophosphate				
c	Guanosine 3′,5′-cyclic monophosphate				
d	1-Methylguanosine				
e	1-Methylguanosine 3′,5′-cyclic monophosphate				
g	gallocatechin				
m	epigallocatechin				
p	Taxifolin-3-sulfate				
q	Epitaxifolin-3-sulfate				
v	Methyldihydroquercetin-3-sulfate				
**Benzopyranones**	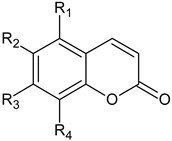	**R_1_**	**R_2_**	**R_3_**	**R_4_**
f	6,8-bissulfooxy-7-hydroxy-2H-1-benzopyran-2-one	H	SO_4_^−^	OH	SO_4_^−^
h	6,7-dihydroxy-8sulfooxy-2H-1-benzopyran-2-one	H	OH	OH	SO_4_^−^
k	7,8-dihydroxy-6-sulfooxy-2H-1-benzopyran-2-one	H	SO_4_^−^	OH	OH
n	8-hydroxy-7-methoxy-6-(sulfooxy)-2H-1-benzopyran-2-one	H	SO_4_^−^	OCH_3_	OH
o	6-methoxy-7-sulfooxy-2H-1-benzopyran-2-one (scopoletin-7-sulfate)	H	OCH_3_	SO_4_^−^	H
r	7-hydroxy-6-methoxy-2H-1-benzopyran-2-one (scopoletin)	H	OCH_3_	OH	H
s	5,6-dimethoxy-7,8-dihydroxy-2H-1-benzopyran-2-one	OCH_3_	OCH_3_	OH	OH
t	7-hydroxy-5,6-dimethoxy-8-sulfooxy-2H-1-benzopyran-2-one	OCH_3_	OCH_3_	OH	SO_4_^−^
u	5,6-dimethoxy-7-sulfooxy-2H-1-benzopyran-2-one (umckalin-7-sulfate)	OCH_3_	OCH_3_	SO_4_^−^	H
w	7-hydroxy-5,6-dimethoxy-2H-1-benzopyran-2-one (umckalin)	OCH_3_	OCH_3_	OH	H

**Table 2:** Antibodies used for Western-blots, immunofluorescence microscopy, and ELISAs. Invitrogen, Waltham, MA, USA; Bio-Techne, Minneapolis, MN, USA; Abcam, Cambridge, UK; BD BioSciences, Franklin Lakes, NJ, USA; and QED-Bioscience Inc., San Diego, CA, USA.

Target Protein	Antibody	Producer	Dilution
Alpha-tubulin	MA1-80017	Invitrogen	1:1000
Claudin4	36-4800	Invitrogen	1:1000
Collagen type-I	NB600-408	Bio-Techne	1:5000
Collagen type-IV	NB120-6586	Bio-Techne	1:2500
Desmocollin2	ab95967	Abcam	1:1000
Desmoglein2	ab150372	Abcam	1:2500
E-cadherin	610181	BD BioSciences	1:5000
Erk1/2 MAPK	Ab180699	Abcam	1:2500
Fibronectin	MA5-11981	Invitrogen	1:1000
Gelsolin	ab109014	Abcam	1:1000
JNK MAPK	ab179461	Abcam	1:5000
P38 MAPK	ab170099	Abcam	1:2500
RV-16	18758	QED-Bioscience Inc.	1:1000

## Data Availability

All original data can be requested from the first author.
